# Delusional Parasitosis Impeding Delivery of Acute Care in a Cancer Patient

**DOI:** 10.3390/diseases6040108

**Published:** 2018-12-05

**Authors:** Ivayla I. Geneva

**Affiliations:** Department of Medicine, State University of New York Upstate Medical University, Syracuse, NY 13210, USA; geneva.ivayla@gmail.com

**Keywords:** delusional parasitosis, delusional infestation, Ekbom’s syndrome, oncologic emergency, the greater good

## Abstract

Taking care of patients with delusional parasitosis can be challenging. This report illustrates the added management complexity and ethical dilemmas surrounding a patient who was newly diagnosed with an incurable lung cancer, while at the same time was suffering from delusional parasitosis. Her delusion was so profound that she blamed flukes for her respiratory failure and refused treatment of her cancer. This paper emphasizes making the right decision with the greater good in mind, even if this meant “yielding” to a patient’s delusion and treating the non-existing parasitosis in order for her to allow us to also treat the cancer.

## 1. Introduction

Delusional parasitosis—the unshakable false belief that parasites have infested the body—is usually thought of in the context of a mental health challenge. It often leads to excessive requests for medical evaluation and treatment, and less commonly to destructive behaviors such as the burning of clothes and even to suicide [1]. This case report describes how delusional parasitosis can do much more than inconvenience the patient and medical provider—the delusion in fact can stand in the way of receiving life-saving treatment for an oncologic emergency. To the best of my knowledge, this is the first report on this topic.

## 2. Case Report

**Figure 1:**
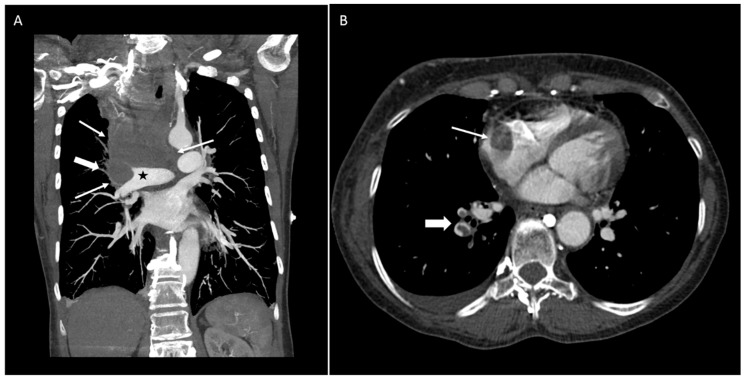
CT image of the thorax from outside the hospital showing (**A**) a large central mediastinal mass, occluding the pulmonary artery and extending into the right atrium, encasing the superior vena cava (SVC), abutting the trachea; and (**B**) pulmonary emboli at the right lower lobe.

A 64-year-old woman with a past medical history of Grave’s disease presented to the hospital complaining of sudden onset of facial and bilateral arm swelling, as well as "bruising" in her neck, abdomen, and arms, all of which she developed over a few days. Further, the patient reported fatigue, neck tightness, and dyspnea on exertion. She denied any other symptoms and the remainder of the full review of systems, including weight loss, was negative. Social history was significant for 40 years of smoking. The patient was hemodynamically stable with good oxygenation on 2 L of oxygen by nasal cannula. Physical exam revealed swelling of the face, neck, and both arms, superficial varicosities on the neck, chest, back, and arms, as well as mild inspiratory stridor. Computed tomography angiography (CTA) of the thorax and neck showed a large central mediastinal mass occluding the pulmonary artery and extending into the right atrium, encasing the superior vena cava (SVC), abutting the trachea but not causing tracheal narrowing (Figure 1A), as well as pulmonary emboli at the right lower lobe (Figure 1B). Given the tobacco abuse history, lung cancer leading to SVC syndrome was suspected, although other malignancies such as lymphoma were in the differential. The patient was started on a heparin drip and the Pulmonary and Hematology-Oncology services were consulted, both of which agreed for the need for tissue diagnosis of the likely malignancy and rapid initiation of treatment. 

A complicating factor in this patient’s care was her adamant belief that the swelling was an allergic reaction to one of the many over-the-counter herbal and homeopathic medications that she was taking to try to rid her body of the “flukes” which she claimed to have seen in her nasal secretions and stool. The patient soon moved on to express the belief that her mediastinal mass was caused by her parasites and demanded treatment for them rather than for the cancer. Review of this patient’s medical record did not yield any evidence for a documented psychiatric disorder or treatment thereof in the past. However, her significant other at bedside revealed that the patient had been preoccupied with the notion of having blood flukes for at least eight years.

After much discussion, the patient agreed upon undergoing biopsy of her mass as long as she was also tested and treated for the parasites. Fine needle aspiration of the right supraclavicular lymph node on day two of admission revealed metastatic small cell carcinoma. On that same day, the patient was transitioned to the ICU due to increasing oxygen requirements necessitating bilevel positive airway pressure ventilation. Radiation–Oncology was consulted and recommended starting radiation treatment; however, the patient was reluctant to do so until the final diagnostic confirmation of cancer on day four. Similarly, she stated that she would refuse to start chemotherapy until she could obtain a second opinion. Not surprisingly, the consulting services deemed it dangerous to postpone treatment initiation given the size and location of the mediastinal mass and the patient’s worsening respiratory status. Due to an enlarging pleural effusion, thoracentesis was performed on the right side on day five and the pleural fluid was negative for malignant cells. Further, a metastatic workup revealed a suspicious lesion at S1 on a nuclear medicine scan that was consistent with Paget’s disease as well as malignancy. An MRI brain scan could not differentiate between small vessel ischemic disease and a possible small metastatic lesion. In the meantime, ova and parasite studies of the patient’s stool, nasal secretions, and lymph node aspirate had returned negative, yet she remained adamant in her decision to refuse chemo-radiation until the parasites were treated. Although there was a plan for an Ethics consultation, it was postponed as the patient changed her mind and underwent the first chemotherapy cycle on days 8 (carboplatin), 9 and 10 (etoposide), and radiotherapy was started on day 10. Ethics was eventually consulted on day 10 and recommended avoiding treatment for unproven parasitosis. Repeat CTA of the thorax on day 12 demonstrated new pulmonary emboli in the left lower lobe, slight increase in the bilateral pleural effusions, and no significant change in the tumor burden. On day 14, the patient decided to change her code status from FULL CODE to DNR/DNI. On day 19, her blood pressure dropped and she required pressor support, was started on a five-day course of cefepime for neutropenic fever, as well as fludrocortisone and hydrocortisone for a few days until the shock resolved. Given her unstable state, further chemotherapy was not advisable. Another complicating factor in the patient’s care were her poor nutritional status, her repeated attempts to drink water despite the danger for aspiration, as well as her increasing agitation and lack of cooperation with the hospital staff. The use of anxiolytics provided only partial relief and the situation continued to escalate to the point where the patient started being verbally abusive to the medical personnel, was refusing medications, radiation therapy, and physical exams. Her respiratory status continued to worsen and she was often heard to voice her wish to die. Therefore, on day 34 a family meeting was held, during which the patient’s poor prognosis was discussed. Although at that time the patient did not wish to pursue comfort care, the prolonged drop in oxygen saturation later that evening was causing her so much discomfort that she decided to make the transition. The patient was pronounced at midnight, from cardiopulmonary failure. Up to the point of death, the patient maintained her belief that the primary cause of her symptoms was a parasitic infestation and that her diagnosis of carcinoma of the lung was “fake”.

## 3. Discussion

Delusional parasitosis, also known as delusional infestation and Ekbom’s syndrome, has been an accepted psychiatric diagnosis since 1894, when it was first described by the dermatologist Georges Thibierge in Paris [2]. This disorder has traditionally been subdivided in two forms: primary and secondary. In the primary form, the belief of parasitic infestation usually manifests itself as an isolated phenomenon of abnormal tactile sensation, with the patient being otherwise mentally healthy [3]. It can be argued that initially this patient was suffering from the primary form, which had been present for several years prior to the current hospitalization. Although she was not having the symptoms of formication, she did describe seeing flukes in her nasal secretions as well as her stool and had been taking several over-the-counter medications to suppress the parasites. However, with the appearance of dyspnea due to the patient’s lung mass, the delusion transformed into the secondary form, where another identifiable disorder caused the symptoms perceived as parasitosis [1,4]. Recommended treatment of this condition is with typical or atypical antipsychotics, but mostly on an off-label basis; and in the case of the secondary form, the underlying medical problem should be targeted. Nevertheless, it should be kept in mind that most patients with delusional parasitosis, including the patient in this case report, deny the diagnosis and are unwilling to undergo treatment [1,5]. Further, in this case report, there was no definitive treatment for the underlying mediastinal tumor.

An important aspect of this case report is the ethical dilemma arising from the patient’s unwillingness to undergo life-saving, or at least life-prolonging, treatment of her metastatic lung cancer. The chemo-radiation was delayed by several days because of her demands for anti-parasitics, despite the lack of evidence for such an infection. Interestingly, the Ethics committee decided that the patient should not be treated for her delusional flukes as long as there was no documented medical need for treatment. However, it should be pointed out that the Ethics committee placed their recommendations only after the patient had given her consent to chemo-radiation. Therefore, it remains unclear for how long, if at all, parasitic treatment should be withheld if a patient would not allow management of the real medical problem. In my opinion, the most urgent medical need should take priority, which in this case was an oncologic emergency. As written above, the psychiatric disorder itself was very challenging to manage and given the constraints of time as the mediastinal mass would grow quickly to compromise the airway, I would be in favor of giving a short course of, e.g., mebendazole, in order to appease the patient and be able to start chemo-radiation promptly. It has been well documented in the literature [1] that patients with delusional parasitosis tend to become apprehensive when challenged with the diagnosis and this was likely a contributing factor in this case where there was an escalating agitation and increasing dissatisfaction with the patient’s medical care. Perhaps, had her delusional parasitosis been treated with an anti-parasitic, she may have been more relaxed and more compliant with medical procedures and medications, which in turn could have prolonged her life expectancy.

Delusional parasitosis remains a challenging condition with no clear treatment guidelines in place. This case report highlights the added complexity in cases where the delusion interferes with important medical care, and I hope it will help raise awareness of this psychiatric entity and spark investigation for optimizing patient care in the presence of this disorder.
